# Evaluation of the Therapeutic Effects of the Hydroethanolic Extract of *Portulaca oleracea* on Surgical-Induced Peritoneal Adhesion

**DOI:** 10.1155/2021/8437753

**Published:** 2021-07-31

**Authors:** Ali Jaafari, Vafa Baradaran Rahimi, Nasser Vahdati-Mashhadian, Roghayeh Yahyazadeh, Alireza Ebrahimzadeh-Bideskan, Maede Hasanpour, Mehrdad Iranshahi, Sajjad Ehtiati, Hamed Rajabi, Mohammadreza Mahdinezhad, Hassan Rakhshandeh, Vahid Reza Askari

**Affiliations:** ^1^Pharmacological Research Center of Medicinal Plants, Mashhad University of Medical Sciences, Mashhad, Iran; ^2^Departments of Pharmacodynamics and Toxicology, School of Pharmacy, Mashhad University of Medical Sciences, Mashhad, Iran; ^3^Department of Cardiovascular Diseases, Faculty of Medicine, Mashhad University of Medical Sciences, Mashhad, Iran; ^4^Department of Anatomy and Cell Biology, School of Medicine, Mashhad University of Medical Sciences, Mashhad, Iran; ^5^Biotechnology Research Center, Pharmaceutical Technology Institute, Mashhad University of Medical Sciences, Mashhad, Iran; ^6^Department of Pharmaceutical Sciences in Persian Medicine, School of Persian and Complementary Medicine, Mashhad University of Medical Sciences, Mashhad, Iran; ^7^Applied Biomedical Research Center, Mashhad University of Medical Sciences, Mashhad, Iran; ^8^Department of Persian Medicine, School of Persian and Complementary Medicine, Mashhad University of Medical Sciences, Mashhad, Iran

## Abstract

**Objective:**

Peritoneal adhesion (PA) is an abnormal connective tissue that usually occurs between tissues adjacent to damaged organs during processes such as surgery. In this study, the anti-inflammatory and antioxidant effects of *Portulaca oleracea* (PO) were investigated against postoperative-induced peritoneal adhesion.

**Methods:**

Thirty healthy male Wistar rats (220 ± 20 g, 6-8 weeks) were randomly divided into four groups: (1) normal, (2) control (induced peritoneal adhesion), and (3) and (4) PO extracts (induced peritoneal adhesion and received 100 or 300 mg/kg/day of PO extract for seven days). Finally, macroscopic and microscopic examinations were performed using different scoring systems and immunoassays in the peritoneal lavage fluid.

**Results:**

We found that the levels of adhesion scores and interleukin- (IL-) 1*β*, IL-6, IL-10, tumour necrosis factor- (TNF-) *α*, transforming growth factor- (TGF-) *β*_1_, vascular endothelial growth factor (VEGF), and malondialdehyde (MDA) were increased in the control group. However, PO extract (100 and 300 mg/kg) notably reduced inflammatory (IL-1*β*, IL-6, and TNF-*α*), fibrosis (TGF-*β*_1_), angiogenesis (VEGF), and oxidative (MDA) factors, while increased anti-inflammatory cytokine IL-10, antioxidant factor glutathione (GSH), compared to the control group.

**Conclusion:**

Oral administration of PO improved postoperational-induced PA by alleviating the oxidative factors, fibrosis, inflammatory cytokines, angiogenesis biomarkers, and stimulating antioxidative factors. Hence, PO can be considered a potential herbal medicine to manage postoperative PA. However, further clinical studies are required to approve the effectiveness of PO.

## 1. Introduction

Abnormal connective fibrous tissues join in the surgical area and cause adhesions between the organs and nearby tissues at nonanatomic locations [1]. Notably, infertility, reintervention, abdominal pain, and intestinal occlusion occur following peritoneal adhesions (PA). The progression rate of PA has related to some risk factors such as surgical trauma, genetic factors, presence of infection, and peritoneal contamination during the surgical operation [2]. In particular, peritonitis is considered one of the main reasons for PA progression mentioned in animal and cellular studies. Furthermore, the duration of operation and type of surgical approach directly are related to the PA formation [3]. In this regard, the open procedure (laparotomy) has been more frequently associated with PA than the laparoscopic approach [4]. Also, in the United States in 1998, the economic burden of adhesions was estimated at around 1437.1 million dollars per year [5]. Some studies indicated the reduction of tissue plasminogen activator (tPA)/plasminogen activator inhibitor-1 (PAI-1) ratio [6], increase of transforming growth factor-beta-1 (TGF-*β*1), tumour necrosis factor-alpha (TNF-*α*) [6], interleukin- (IL-) 6 [7], vascular endothelial growth factor (VEGF) [8] and cyclooxygenase (COX), and inhibition of proteolytic enzymes (e.g., matrix metalloproteases (MMPs)) [9], caused to PA development [10]. In addition, further research also proved that upregulation of inducible nitric oxide synthase (iNOS) [9], stress oxidative markers, and myeloperoxidase (MPO) promoted PA progression and development [10]. These alterations are considered underlying factors for the generation of collagen type-1 and PA development [11]. As primary cells involving PA development, phagocytic and secretory activities of macrophages are increased after five days from surgical procedure and injury by rolling the immune system. Indeed, the surface of the injured area was renewed by macrophages that provide new mesothelial layers with the help of fibroblast cells, usually three to five days after surgical injury [12]. The formation and development of the fibrins were inhibited by chemical agents such as glucocorticosteroids, calcium channel blockers, nonsteroidal anti-inflammatory drugs (NSAIDs), antibiotics, histamine antagonists, and fibrinolytic agents [13]. However, they have no enough effectiveness and efficacy for the prevention or treatment of PA.

The therapeutic advantages of herbal medicine were considered in some research because of its availability, possible efficacy, and safety [14]. *Portulaca oleracea* L. (PO) is a warm-climate herbaceous, namely, “purslane” in the USA and Australia, a famous rig in Egypt, pigweed in England, pourpier in France, Ma-Chi-Xian in China, and Qurfeh in Iran [15, 16]. Also, it is the main source of phosphorus, calcium selenium, manganese, iron [17], and omega-3 fatty acids that promote immune function [16, 18]. There are several reports of benefits of the PO plant, including attenuating effects on cancers, coronary artery disease, hypertension, and inflammatory and autoimmune disorders [16, 19]. Experimentally, it has been shown that the plant has several active constituents, including monoterpenes (portulosides A and B), diterpenes (portulene), *β*-amyrin type triterpenoids, and vitamin A [20]). Besides, it contains *α*-tocopherol, ascorbic acid, B-complex vitamins (niacin, pyridoxine, and riboflavin) [21], and amino acids (leucine, lysine, phenylalanine, methionine, isoleucine, proline, cysteine, valine, threonine, and tyrosine) [16, 22]. Moreover, further *in vivo* and *in vitro* studies represented neuroprotective [18], antidiabetic [23], antioxidant [24], anticancer [25], antiulcerogenic [26], and hepatoprotective [15] effects of PO. However, there is no study evaluating the effectiveness of the oral administration of PO on preventing surgical-induced peritoneal adhesion. Therefore, in the present study, we investigated the protective effects of PO against the surgical-induced peritoneal adhesion in a rat model.

## 2. Materials and Methods

### 2.1. Drugs and Chemicals

Ethanol was prepared from Sigma-Aldrich Chemical Co. (St. Louis, MO, USA). Normal saline was purchased from the Samen® pharmacy factory (Iran). Ketamine and xylazine were obtained from ChemiDaru Company (Iran). Enzyme-linked immunosorbent assay (ELISA) kits, including VEGF, IL-1*β*, IL-6, TGF-*β*, IL-10, and TNF-*α*, were purchased from IBL International® Company (Switzerland), and malondialdehyde (MDA), nitric oxide (NO), and glutathione (GSH) kits were prepared from ZellBio Company (Germany).

#### 2.1.1. Plant Material and Preparation of the Extract

PO (herbarium No. 12-1615-240) was prepared from Mashhad, Khorasan Razavi Province, Iran, in Jan 2020. First, the aerial parts of the plant were freshly prepared then washed and dried in the shadow (25°C). After the complete drying of the plant, 100 g of the plant was powdered using a mill. Next, the extract was prepared by the maceration method using 800 ml of 70% *v*/*v* ethanol/water solution for the next 72 h. Afterwards, the obtained liquid extract was concentrated using a rotary evaporator at 40°C, which yields a solid powder (20% *w*/*w* of dried powder) [27–29]. This powder is stored in the freezer at -20°C until experimenting. Finally, the extract was dissolved in normal saline containing 5% *v*/*v* Tween 80 [30].

#### 2.1.2. Liquid Chromatography-Mass Spectrometry (LC-MS) Apparatus

The LC-MS analysis was performed using an AB SCIEX QTRAP (Shimadzu) liquid chromatography coupled with a triple quadrupole mass spectrometer. Liquid chromatography separation was performed on a Supelco C18 (15 mm × 2.1 mm × 3 *μ*m) column. The analysis was done at a flow rate of 0.2 ml/min. The binary mobile phase consisted of A: 0.1% formic acid in water and B: 0.1% formic acid in acetonitrile. The gradient analysis started with 10% of B, isocratic conditions were maintained for 10 min, gradually turned to 30% B over 20 min, gradually increased to 80% B over 30 min, and held at 80% B for 10 min, and the system was turned to the initial condition of 10% B in 5 min. Finally, the system was reequilibrated over 5 min. The mass spectra were acquired in a range of 100 to 1700 within the 80-minute scan time. The positive electrospray ionisation (ESI) mode was applied for the mass spectrometer. Mass feature extraction of the acquired LC-MS data and maximum detection of peaks was done using the *MZ*mine analysis software package, version 2.3.

### 2.2. Experimental Animals and Ethical Statements

The investigation was performed with male Wistar rats weighing 200-250 g obtained from the animal laboratory of Faculty of Medicine, Mashhad University of Medical Sciences, Mashhad, Iran. The animals were preserved in the institute animal house at 24 ± 1°C with a 12/12 h light-dark cycle for one week before and through the experiment with free access to water and food. All animal procedures were carried out in compliance with the National Institutes of Health's Guide for the Use and Care of Laboratory Animals guidelines of institutional guidelines. Moreover, the National Institute for Medical Research Development ethical committee approved all procedures involving animals based on the policies of animal experiments and care (Ethical approval code: 971254, Approval date: 2018–07–01, Approval ID: IR.NIMAD.REC.1397.084).

### 2.3. Model Induction and Grouping and Interventions

To generate abdominal adhesions, the sterilised gauze was used to induce peritoneal abrasion and adhesion. First, all rats were anaesthetised with intraperitoneal (i.p.) injection of 100 mg/kg ketamine, 10 mg/kg xylazine, and 3 mg/kg acepromazine, and then, rats' abdominal hair was carefully shaved and then washed with 70% *v*/*v* ethanol [30]. Next, the sterilised gauze was repeatedly contacted to the peritoneum wall until the appearance of the faded purple spot on the peritoneum. Then, the peritoneal tissue was sutured with nonabsorbable sutures No. 4.0. After that, this area was disinfected with a few drops of chloramphenicol [31–33]. At the end of the surgery, the rats received cefazolin 300 mg/kg (i.m.) to prevent wound infection [32, 33] and then were transferred to the recovery room for grouping and interventions for seven days. The surgery procedure was prolonged to a maximum of ten min. It should be noted that the gavage of groups was performed on the first day after the surgery.

Thirty healthy male Wistar rats (220 ± 20 g, 6-8 weeks) were randomly divided into four groups, as described below: group one, normal (six rats without surgical procedures); group two, control (eight rats induced peritoneal adhesion and gavaged with the vehicle of PO extract for seven consecutive days); group three (low dose, eight rats induced peritoneal adhesion and received 100 mg/kg/day of PO extract for seven consecutive days); group four (high dose, eight rats induced peritoneal adhesion and received 300 mg/kg/day of PO extract for seven consecutive days). The doses of PO were selected according to the preliminary evaluation.

### 2.4. Assessment of Adhesion Grade

The rats' laparotomy was done on the 8^th^ day after the surgery. The peritoneal adhesion grades were scored via the two scoring systems (Tables 1 and 2) with two independent researchers blinded on the procedure and the grouping.

### 2.5. Histological Evaluation

In this study, paraffin-embedded histological sections were stained by Masson's trichrome staining to assess the extent and distribution of fibrosis in rats' peritoneal tissue as described by the manufacturer (Sigma-Aldrich) [34, 35]. In addition, to prepare the peritoneal tissue sample, after removing formalin and washing with distilled water three times, the tissues were transferred to different concentrations of alcohol (50-100% *v*/*v*) for some minutes. Tissue sections were observed with magnifications of 4x, 20x, and 40x using a Nikon E-1000 microscope (Japan) under bright-field optics and photomicrographed using Easy Image 1 (Bergström Instrument AB, Sweden).

### 2.6. Total Protein Measurement Method

The Bradford protein assay was performed to quantify the total protein concentration in a sample [36]. For this reason, first, the Coomassie Brilliant Blue G-250 dye (10 mg) was dissolved in 50 ml ethanol 96%. Then, phosphoric acid 85% (10 ml) was added and the volume of the solution was increased to 100 ml. Next, bovine serum albumin (4 mg/ml) solution was prepared as a standard curve. Then, after sample pouring (20 *μ*l), a Bradford reagent (200 *μ*l) was added to the 96-well microplate. Finally, the light absorption was read at 595 nm with a microplate reader after 5 minutes.

### 2.7. Evaluation of Inflammatory, Angiogenesis, and Fibrosis Biomarkers

According to the manufacturer's instruction, as indices of inflammation, the levels of TNF-*α*, IL-1*β*, and IL-6 were measured in peritoneal lavage fluid using ELISA kits [37, 38]. In addition, the levels of VEGF, as an angiogenesis marker, TGF-*β*, as a fibrosis factor, and IL-10, as an anti-inflammatory and suppressive cytokine, were also measured in the peritoneal fluid sample by ELISA kits according to the manufacturer's instruction [31, 39].

### 2.8. Measurement of Oxidant and Antioxidant Parameters

The levels of malondialdehyde (MDA), as an oxidant marker, and glutathione (GSH), as an antioxidant marker, were measured in the peritoneal fluid using commercially available biochemistry kits [31, 38, 40].

### 2.9. Statistical Analysis

Data were analysed using GraphPad Prism software (version 6.01) and represented as mean ± SD and median ± interquartile range (IQR), according to the nature of parametric or nonparametric data, respectively. A one-way analysis of variance (ANOVA) was performed with Tukey's Kramer multiple comparison posttest for parametric data. However, for the nonparametric data (adhesion scores), a Kruskal-Wallis' test was performed with Dunn's multiple comparison post hoc test. *P* values (*P*) when lower than 0.05 were considered statistically significant [28, 39, 40].

## 3. Results

### 3.1. LC-MS Analysis of PO Extract

**Figure 1:**
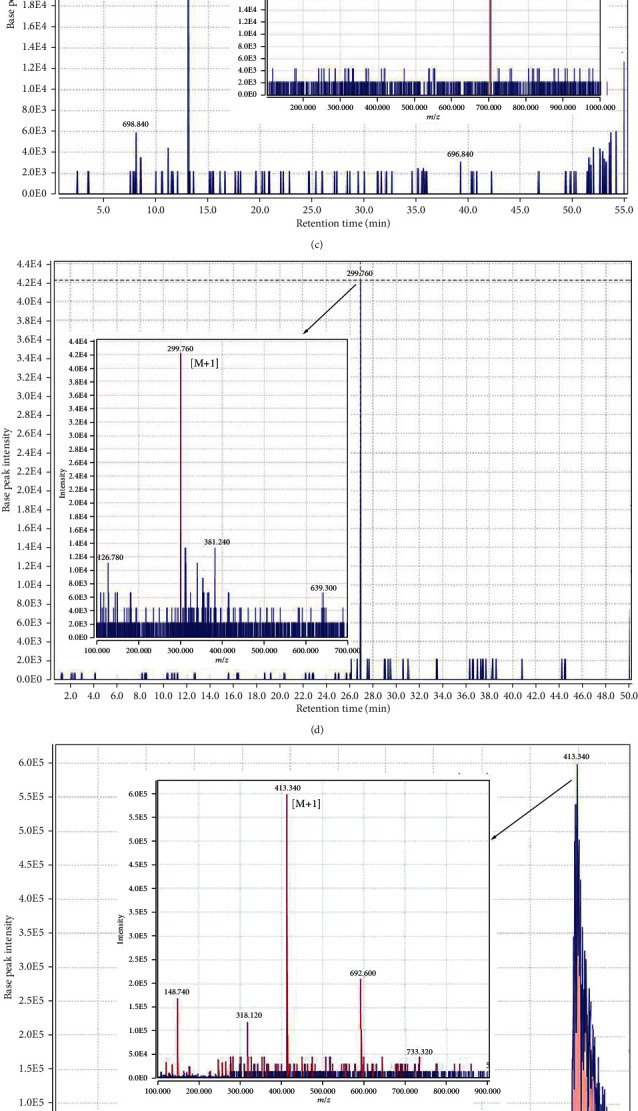
(a) The total ion chromatogram of *Portulaca oleracea* extract; (b) chromatogram of dopa and corresponding mass adduct, [M + 1], at *m*/*z* 198.120; (c) chromatogram of oleraceins D and corresponding mass adduct, [M + 1], at *m*/*z* 696.84; (d) chromatogram of portulacanone D and corresponding mass adduct, [M + 1], at *m*/*z* 299.76; (e) chromatogram of friedelane and corresponding mass adduct, [M + 1], at *m*/*z* 413.34; (f) chromatogram of riboflavin and corresponding mass adduct, [M + 1], at *m*/*z* 376.62; (g) chromatogram of aurantiamide acetate and corresponding mass adduct, [M + 1], at *m*/*z* 445.8.

In total, 30 compounds were identified in the hydroethanolic extract of the aerial parts of PO, mainly including alkaloids, flavonoids, terpenoids, and vitamins. Data concerning the identification of the compounds are represented in Table 3. The total ion chromatogram of PO extract is shown in Figure 1(a). In addition, the MS spectral data were compared with the reported compounds in some previous literature. Examples of extracted ion chromatograms from the total ion chromatogram and its related mass are represented in Figures 1(b)–1(g). Alkaloids are one of the important chemicals found in PO, including dopa, noradrenalin, and oleraceins A, B, C, and D (cyclodopa alkaloids). Moreover, PO contains monoterpenes (portulosides A), diterpenes (portulene), ascorbic acid, α-tocopherol, and riboflavin.

### 3.2. The Effects of PO Extract on Peritoneal Adhesion (PA) Scoring

**Figure 2:**
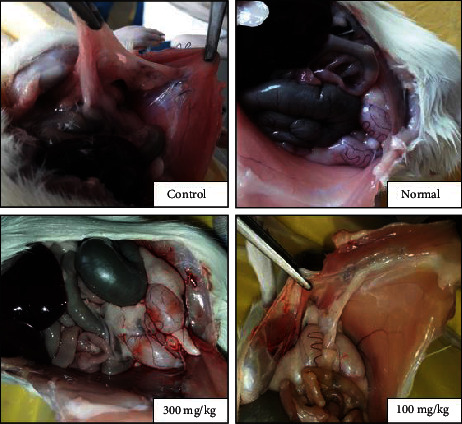
Macroscopic evaluation of PA bands in normal, control, and PO at doses of 100 and 300 mg/kg groups.

**Figure 3:**
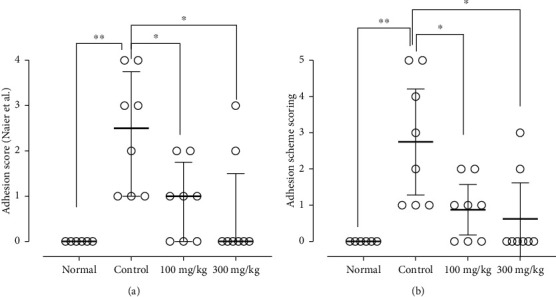
The effects of different doses of PO on adhesion scores evaluated by Naier et al. (a) and Adhesion Scoring Scheme (b) scoring systems; data were presented as median ± interquartile range, IQR (*n* = 8 for all groups except normal as 6). ^∗^*P* < 0.05 and ^∗∗^*P* < 0.01.

A macroscopic evaluation of PA scores was performed at the end of the experiment (Figure 2). We found that the PA scores were significantly increased in the control group compared to the standard group (*P* < 0.01 for both scoring systems, Figures 3(a) and 3(b)). Conversely, both doses of PO (100 and 300 mg/kg) markedly abolished the PA scores compared to the control group (*P* < 0.05 for both cases and scoring systems).

### 3.3. The Effects of PO Extract on Histopathological Alteration of Peritoneal Fibrosis

**Figure 4:**
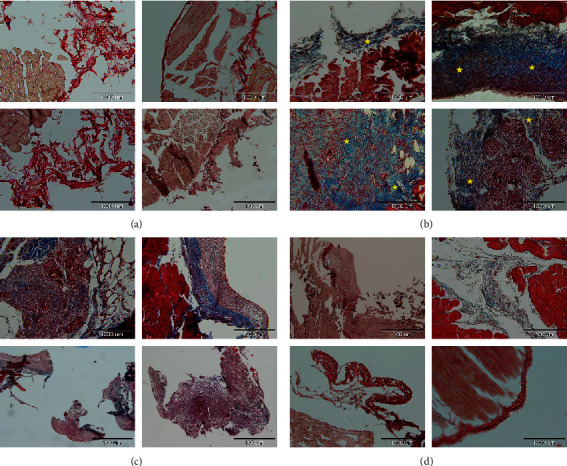
The effects of different doses of PO on adhesion formation and collagen deposition by histopathological evaluation using Masson's trichrome staining; blue colour intensities (marked with yellow stars) represent fibrosis and collagen deposition. Pathological imaging; (a) normal group, (b) control group, (c) PO at the dose of 100 mg/kg, and (d) PO at the dose of 300 mg/kg. The highest rate of tissue fibrosis (blue colour) and collagen deposition was observed in the control group (b) compared to the normal group (a), which had the lowest fibrosis rate. Conversely, the blue colour intensities as a marker of fibrosis and collagen deposition in both doses of the extract groups (100 and 300 mg/kg) were significantly decreased compared to the control group (c, d).

The histopathological study was performed using Masson's trichrome staining to evaluate the rate of peritoneal fibrosis. As shown in Figure 4, the highest rate of tissue fibrosis (blue colour) and collagen deposition was observed in the control group (Figure 4(b)) compared to the normal group (Figure 4(a)), which had the lowest fibrosis rate. In contrast, the blue colour intensities as a marker of fibrosis and collagen deposition in both doses of the extract groups (100 and 300 mg/kg) were significantly decreased compared to the control group (Figures 4(c) and 4(d)).

### 3.4. The Effects of PO Extract on Inflammatory and Anti-Inflammatory Biomarkers

**Figure 5:**
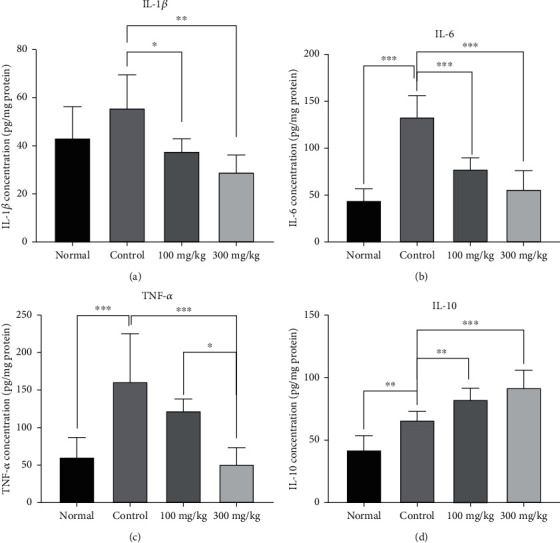
The effects of different doses of PO on the peritoneal lavage levels of (a) IL-1*β*, (b) IL-6, (c) TNF-*α*, and (d) IL-10; data were presented as mean ± SD (*n* = 8 for all groups except normal as 6). ^∗^*P* < 0.05, ^∗∗^*P* < 0.01, and ^∗∗∗^*P* < 0.001.

Our results indicated that the levels of inflammatory mediators, including IL-6 (*P* < 0.001, Figure 5(b)) and TNF-*α* (*P* < 0.001, Figure 5(c)), and anti-inflammatory cytokine IL-10 (*P* < 0.01, Figure 5(d)) were significantly increased in the control group compared to the normal group. However, the level of IL-1*β* was greater than the normal group, but this increment was not statistically significant (Figure 5(a)). Administration of PO for seven consecutive days notably reduced the levels of IL-1*β* (100 mg/kg, *P* < 0.05, and 300 mg/kg, *P* < 0.001, Figure 5(a)), IL-6 (*P* < 0.001 for 100 and 300 mg/kg, Figure 5(b)), and TNF-*α* (300 mg/kg, *P* < 0.001, Figure 5(c)) and significantly elevated the level of IL-10 (100 mg/kg, *P* < 0.01, and 300 mg/kg, *P* < 0.001, Figure 5(d)) in the peritoneal lavage fluid, compared to the control group.

### 3.5. The Effects of PO Extract on Fibrosis and Angiogenesis Parameters

**Figure 6:**
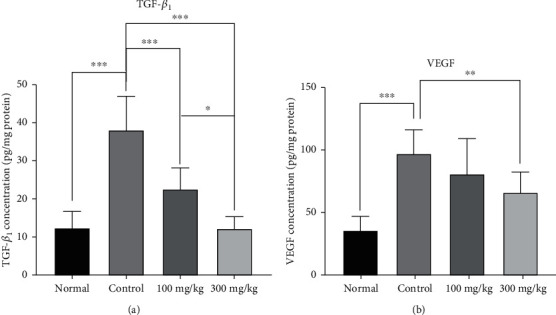
The effects of different doses of PO on the peritoneal lavage levels of (a) TGF-*β* and (b) VEGF; data were presented as mean ± SD (*n* = 8 for all groups except normal as 6). ^∗^*P* < 0.05, ^∗∗^*P* < 0.01, and ^∗∗∗^*P* < 0.001.

Following the PA, the levels of TGF-*β*1 as a fibrotic factor and VEGF as an angiogenesis factor were significantly enhanced in the control group compared to the normal group (*P* < 0.001 for both cases, Figures 6(a) and 6(b)). In contrast, the use of 300 mg/kg/day PO significantly mitigated the levels of TGF-*β*1 and VEGF compared to the control group (*P* < 0.001 for both cases, Figures 6(a) and 6(b)). However, 100 mg/kg/day of PO could significantly attenuate the level of TGF-*β*1 compared to the control group (*P* < 0.001, Figure 6(a)). Moreover, the results indicated that the suppressive effect of 300 mg/kg/day PO on TGF-*β*1 level was more than 100 mg/kg/day PO (*P* < 0.05, Figure 6(a)).

### 3.6. The Effects of PO Extract on Oxidant and Antioxidant Levels

**Figure 7:**
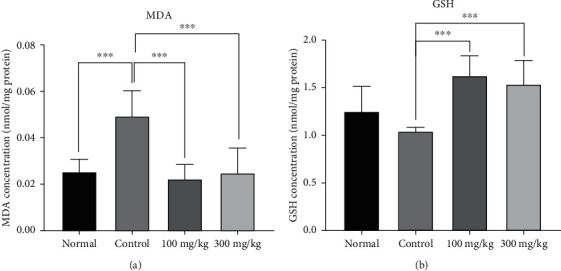
The effects of different doses of PO on the peritoneal lavage levels of (a) MDA and (b) GSH; data were presented as mean ± SD (*n* = 8 for all groups except normal as 6). ^∗∗^*P* < 0.01 and ^∗∗∗^*P* < 0.001.

The results revealed that the MDA level was significantly elevated in the control group compared to the normal group (*P* < 0.001, Figure 7(a)). Nevertheless, 100 and 300 mg/kg/day PO administration remarkably abolished the MDA levels compared to the normal group (*P* < 0.001 for both cases, Figure 7(a)). Following the PA induction, we observed that the level of GSH slightly decreased in the control group in comparison to the normal group (Figure 7(b)). However, administration of PO (100 and 300 mg/kg/day) considerably augmented the GSH levels in peritoneal lavage fluid compared to the control group (*P* < 0.001 for both cases, Figure 7(b)).

## 4. Discussion

To the best of our knowledge, this is the first study evaluating the effects of oral administration of PO extract on PA in a rat model. As a result, we found that PO at both doses significantly reduced the adhesion formation score by lowering the inflammatory cytokines (IL-1*β*, IL-6, and TNF-*α*), increasing the anti-inflammatory cytokine IL-10, and suppressing TGF-*β*1 and VEGF as fibrotic and angiogenesis factors, respectively. Moreover, PO extract regulated the imbalanced oxidant/antioxidant markers by lowering MDA level as a marker of lipid peroxidation and enhancing GSH level as an antioxidant system's reservoir.

The peritoneum is a thin and delicate membrane covering the abdominal cavity and protects and structures internal organs. Some pathologic processes, including ischemia, haemorrhage, endometriosis, infections, trauma, and surgical procedures, cause PA generation in the overwhelming majority of patients [12, 41]. Nevertheless, its effects in everlasting fibrinous adhesions are influenced by the integrity of the fibrinolytic system [42]. Several investigations showed that inflammatory cytokines, including TNF-*α*, IL-6, and IL-1*β*, are the primary reasons for PA generation [30, 43, 44]. In this regard, our investigation also revealed that the levels of the inflammatory cytokines (TNF-*α*, IL-6, and IL-1*β*) were meaningfully increased in the control group following postoperational-induced PA. In contrast, PO at both doses markedly abolished these inflammatory markers. In line with our findings, it has been reported that administration of PO (200 and 400 mg/kg/day for four weeks) significantly abrogated systemic oxidative stress (MDA) and inflammatory markers (TNF-*α* and IL-6) in streptozotocin-induced diabetic rats [45].

Transforming growth factor-*β* (TGF-*β*), as a pleiotropic cytokine, modulates the immune system activity of T cells and regulates inflammation [30, 46]. VEGF is the essential angiogenesis activator. It also induced leakage and proliferating in endothelial cells to the adhesion site and generated new blood vessels [47, 48]. The Cahill et al. investigation results revealed that suppressing the VEGF via VEGF monoclonal antibody (bevacizumab) mitigates PA formation in mice [49]. In fact, both VEGF and TGF-*β*1 expression levels are increased during the PA process [47, 48, 50]. Our present study demonstrated that VEGF and TGF-*β*1 levels were significantly elevated in the control group following the postoperational-induced PA compared to the normal group accordingly. In immunohistological research, treatment of mice with PO (300 mg/kg/day, p.o., for ten weeks) revealed that it could reduce the expression levels of advanced glycation end products (AGE), TGF-*β*1, and intercellular adhesion molecule- (ICAM-) 1 in diabetic nephropathy through suppression of renal fibrosis and inflammation in diabetic *db*/*db* mice [50]. Similarly, PO extract (300 mg/kg/day, p.o., for ten weeks) reduced vascular-related adhesion molecules such as endothelial vascular cell adhesion molecule-1 (VCAM-1), intercellular adhesion molecule-1 (ICAM-1), and E-selectin in diabetic *db*/*db* mice [51]. Moreover, we also exhibited that the PO extract (100 and 200 mg/kg, p.o.) inhibited lung inflammation by downregulating TNF-*α*, IL-6, IL-*β*, TGF-*β*, and PGE_2_ levels and upregulating the expression level of IL-10 [29]. In addition, it was found that PO abolished the lung wet/dry ratio that was an index of oedema and improved the levels of MDA, MPO, WBC, thiol group formation, and CAT and SOD activities compared with the LPS group [29].

One of the essential parameters of PA generation is oxidative stress [52]. Hence, in the current study, we investigated the levels of MDA and GSH as oxidant/antioxidant markers. Our data emphasised that PO meaningfully reduced the level of MDA and enhanced the GSH level following the postoperational-induced PA. Furthermore, it has been reported that the PO extract could elevate the SOD and CAT levels, while downregulating the MDA level in lipopolysaccharide- (LPS-) induced acute lung injury rats [37]. In this context, another study indicated that the PO extract improves SOD and GSH levels and prevents MDA and IL-6 levels in the STZ-induced diabetic rats [45]. Thus, these studies can support our findings on the antioxidant activity of PO that led to the reduction of adhesion formation.

In conclusion, our investigation represented that oral administration of PO improved postoperational-induced PA via alleviating the oxidative factors, fibrosis, inflammatory cytokines, angiogenesis biomarkers, and stimulating antioxidative factors. Hence, PO can be considered a potential herbal medicine to manage postoperative PA. However, further clinical studies are required to approve the effectiveness of PO.

## Figures and Tables

**Table 1:** Scoring system for peritoneal adhesion according to the Nair et al. criteria [53].

Grade	Description of adhesive bands
0	The complete absence of adhesions
1	Only one band of adhesions among the viscera or between one viscera and the abdominal wall
2	Two bands: among viscera's or from viscera to abdominal wall
3	More than two bands: among viscera or from viscera to the abdominal wall or all intestine making a mass without adhesion to the abdominal wall
4	Viscera adhered directly to the abdominal wall, independent of the number and the extension of adhesion bands

**Table 2:** Scoring system for peritoneal adhesion according to Adhesion Scoring Scheme [44, 53].

Grade	Description of adhesive bands
0	Absence of adhesions
1	A thin layer adhesion
2	More than a thin layer adhesion
3	Thick adhesive tissue attached to the surgical site
4	Thick adhesive tissue attached to different areas of the abdomen
5	Thick adhesive tissue containing blood vessels or too much adhesive tissue or organ adhesive tissue

**Table 3:** Peak assignment of metabolites in the hydroethanol extract of PO using LC-MS in the positive mode.

Peak No.	Compound identification	*t*_R_ (min)	M + H (*m*/*z*)	Ref.
1	Portulacanone D	26.9	299.76	[25]
2	Noradrenaline	37.0	170.7	[54]
3	Dopa	15.0	198.12	[55]
4	Oleraceins A	62.5	504.66	[55]
5	Oleraceins B	9.5	533.76	[55]
6	Oleraceins C	64.1	666.06	[55]
7	Oleraceins D	13.1	696.84	[55]
8	Adenosine	19.8	268.8	[55]
9	(3R)-3,5-Bis(3-methoxy-4-hydroxyphenyl)-2,3-dihydro-2(1H)-pyridinone	89.3	342.36	[56]
10	Aurantiamide acetate	36.4	445.8	[57]
11	Cyclo(L-tyrosinyl-L-tyrosinyl)	67.7	327.24	[57]
12	Portuloside A	72.2	332.22	[58]
13	Portulene	66.3	337.02	[15]
14	Lupeol	66.5	427.5	[15]
15	(3S)-3-O-(*β*-D-Glucopyranosyl)-3,7-dimethylocta-1,6-dien-3-ol	67.8	318.12	[59]
16	Friedelane	54.9	413.34	[60]
17	Quercetin	39.4	303.18	[61]
18	Myricetin	55.1	318.24	[61]
19	Genistin	65.4	433.20	[62]
20	Indole-3-carboxylic acid	77.8	162.90	[25]
21	Palmitic acid	62.2	256.14	[63]
22	Stearic acid	37.8	285.18	[63]
23	Caffeic acid	65.8	181.08	[64]
24	Riboflavin	35.0	376.62	[21]
25	Vitamin C	28.5	177.00	[21]
26	*α*-Tocopherol	67.1	431.22	[63]
27	Hesperidin	76.8	611.58	[65]
28	Portulacerebroside A	64.6	843.18	[60]
29	*β*-Sitosterol	48.7	415.32	[15]
30	*β*-Carotene	37.5	538.74	[63]

## Data Availability

The data used to support the findings of this study are available from the corresponding author upon reasonable request.
